# When “Advances” Become Substitutes for Access: A Systems Critique of Children’s Dentistry in NHS England and the Normalisation of Extraction, Containment, and Planned Tooth Loss

**DOI:** 10.3390/children13020263

**Published:** 2026-02-13

**Authors:** Ziad D. Baghdadi

**Affiliations:** 1 TopSmiles Pediatric Dentistry and Orthodontics, Winnipeg, MB R2M3A4, Canada; zalbaghdadi@atsu.edu or drziadeddin.albaghdadi@gmail.com; 2 Butterfly Dental Group, Winnipeg, MB R3Y1P5, Canada

**Keywords:** NHS dentistry, paediatric oral health, early childhood caries, general anaesthesia, health inequalities, Hall Technique, silver diamine fluoride, water fluoridation, MIH, dental contract, implementation

## Abstract

**Highlights:**

**What are the main findings?**

- **Innovation is being “captured” by scarcity:** In NHS England, constrained access and continuity can turn minimally invasive/biological options (Hall Technique, SDF) from *bridges to definitive care* into *system-stable endpoints*—a form of implementation drift where the service environment quietly rewrites what “evidence-based” means in practice.
- **An ethical inversion is emerging:** Tools meant to *prevent irreversible outcomes* are increasingly deployed in pathways that still culminate in irreversible outcomes (hospital extractions; planned loss of compromised first permanent molars), so “advance” functions less as progress and more as a mechanism that normalises late-stage, efficiency-driven decision-making.

**What is the implication of the main finding?**

- **Efficacy is not the same as progress:** Paediatric dental “advances” should be judged by whether they *change system endpoints* (earlier attendance, sustained prevention, fewer crisis referrals/extractions), not merely whether they work in trials—because a broken pathway can convert effective interventions into elegant substitutes for access.
- **Policy must build “bridge rules,” not just toolkits:** Commissioning should hardwire recall intervals, escalation triggers, and guarantees of restorative/specialist capacity (especially for MIH/first permanent molars) so that biologic management and selective extraction guidance remain *patient-centred choices* rather than *capacity-driven defaults*.

**Abstract:**

**Background**: England is a high-income country with a predominantly publicly funded health system organised around the National Health Service (NHS). Yet children’s oral health outcomes continue to reflect a persistent access and prevention gap, with late presentation and hospital-based extractions remaining common. **Objective**: To present a policy-facing, evidence-informed critique of how structural constraints in NHS dentistry shape paediatric clinical pathways—often converting “advances” (biological caries management, silver diamine fluoride, and planned extraction pathways for compromised permanent molars) into compensations for service failure rather than patient-centred progress. **Methods**: Narrative commentary drawing on UK official statistics and major policy reports, alongside key clinical trials and evidence syntheses relevant to contemporary paediatric dentistry. **Results**: The dominant failure mode is not a lack of clinical tools but impaired delivery: restricted access to routine NHS dentistry, contract and workforce pressures, and unequal prevention coverage. These pressures correlate with crisis-led care (including extractions under general anaesthesia) and can distort how minimally invasive/biological interventions are used—functioning as endpoints rather than bridges to definitive care. In parallel, guidance for compromised first permanent molars (including those affected by MIH) risks being operationalised as an “efficiency pathway” when restorative capacity is constrained. **Conclusions**: In NHS England, paediatric dental “advances” cannot be judged solely by trial efficacy; they must be evaluated within a delivery system that currently selects for late-stage, irreversible outcomes. A credible “advances” agenda requires contract reform, workforce retention, prevention at scale, and explicit safeguards against the normalisation of extraction-only trajectories.

## 1. Introduction

England’s health system is largely “the NHS” in terms of financing and access expectations: the UK Health Accounts show government spending as the principal mode of healthcare financing, accounting for 81.3% of total healthcare expenditure in 2024 (vs. 79.0% in 2019), with out-of-pocket spending at 14.6% in 2024. Importantly, the Health Accounts also caution that their definition is broader than “NHS spending” per se, so the figures should be interpreted as a system-financing overview rather than a direct NHS budget line-item [1].

This public-financing dominance creates a moral and political premise: a wealthy, publicly funded system should not routinely allow preventable childhood dental disease to progress to crisis care and irreversible loss. Yet children’s dentistry in England increasingly exposes a mismatch between national capacity and service delivery—where “what can be done” clinically is eclipsed by “what can be reached” operationally.

A further nuance is that “NHS care” is not always delivered by NHS-owned providers. The UK government has highlighted substantial use of independent-sector providers for NHS-funded activity (e.g., 6.15 million NHS appointments, tests and operations delivered by the independent sector in one cited annual period) [2]. This reinforces a key point: financing and entitlement can remain NHS-based even while delivery is fragmented across provider types.

In this article, “advances” in paediatric dentistry are examined through an implementation lens: when access collapses, and prevention is inconsistently delivered, clinical innovations risk being reinterpreted into system-compatible doctrines—containment instead of cure, temporisation instead of timely care, and extraction instead of rescue.

### Evidence Selection and Limitations

This article is a narrative perspective rather than a systematic review. Evidence was selected with priority given to UK official statistics, major national policy reports, and landmark clinical trials or evidence syntheses relevant to paediatric dentistry and service delivery. The discussion does not capture all local commissioning heterogeneity, unpublished operational data, or the full uncertainty surrounding population-level interventions (including differential impacts on inequalities). The aim is therefore an evidence-informed systems critique, not a comprehensive synthesis.

## 2. The Core System Defects Shaping Children’s Dental Outcomes in NHS England

### 2.1. Access Failure: The Front Door Is Partially Closed

A primary defect is restricted access to routine NHS dental care, particularly for households without an established NHS dentist. In the Office for National Statistics (ONS) Health Insight Survey dataset (reported by the British Dental Association), 96.9% of respondents who did not have a dentist and who tried to access NHS dental care reported being unsuccessful [3,4]. Reduced routine access translates into missed preventive contacts (risk assessment, fluoride varnish, anticipatory guidance) and delayed disease detection. NHS dental statistics show substantial activity but also incomplete preventive coverage: in England in 2023/24, 6.6 million child patients were seen in the 12 months to March 2024, and fluoride varnish was recorded in 56% of children’s courses of treatment [5]. Without continuity, early dental caries can progress unchecked until pain or infection forces urgent presentation, shifting management from preventive and minimally invasive care toward crisis-led intervention.

### 2.2. Contract and Workforce Dynamics: Incentives Misaligned with Prevention and Complexity

The National Audit Office (NAO) investigation into the NHS dental recovery plan describes how NHS dentistry in England is commissioned via contracts built around Units of Dental Activity (UDAs) and highlights ongoing structural problems in delivery and recovery planning [6]. When time-intensive paediatric prevention and behaviour support are not operationally protected, a predictable shift occurs: complex child-centred care becomes harder to provide at scale, and the system drifts toward throughput and crisis management.

### 2.3. The Downstream Burden: Hospital Tooth Extractions Remain Common

The Office for Health Improvement and Disparities (OHID) reports 49,112 hospital tooth extraction episodes for 0–19-year-olds in England in the financial year ending 2024, of which 30,587 (62%) had a primary diagnosis of tooth decay (i.e., dental caries) [7]. These indicators describe episodes of care rather than unique individuals, and anaesthetic method is not recorded; however, OHID notes that the majority of episodes are likely to involve general anaesthesia [7]. Regardless of anaesthesia, the volume of hospital extraction activity signals late-stage presentation and a pathway that too often terminates in irreversible intervention.

### 2.4. Inequalities: Deprivation Is Biologically Expressed as Dental Disease

England’s child oral health data show a persistent social gradient. The 2024 National Dental Epidemiology Programme survey of 5-year-olds reports that children in the most deprived areas had 2.7 times the prevalence of obvious dentinal caries compared with those in the least deprived areas (32.2% vs. 13.6%) [8]. For a preventable disease, this postcode gradient points to unequal exposure to prevention and unequal access to timely care, with direct implications for commissioning, prevention delivery at scale, and equity-focused access standards.

### 2.5. Prevention Tools Exist but Are Not Consistently Delivered or Scaled

National prevention guidance exists (e.g., *Delivering Better Oral Health*), including routine preventive measures such as fluoride varnish and age-appropriate fluoride toothpaste recommendations [9]. The issue is not that prevention is unknown; it is that prevention is not reliably *delivered* across populations most at risk—especially when access to routine care is itself unstable.

## 3. When Extraction Under General Anaesthesia Becomes “Normal”

General anaesthesia (GA) is sometimes clinically necessary in paediatric dentistry. The ethical and systems concern is how often severe dental caries presents only after delayed access, making hospital-based extraction a common endpoint rather than a last resort after early prevention and timely conservative treatment. National indicators report hospital tooth extraction episodes for 0–19-year-olds; these are episodes (not unique individuals), and anaesthetic method is not captured, although OHID notes that the majority of episodes are likely to involve GA [7]. In this perspective, “GA extraction” is therefore used as a clinically common (but not identical) proxy for severe late presentation and crisis-led care.

OHID’s hospital extraction indicator illustrates the scale of secondary care involvement and its persistence over time [5]. Separately, the Parliamentary Office of Science and Technology (POST) has highlighted large numbers of hospital admissions for extraction of caries-affected teeth and associated NHS costs, framing childhood dental extractions as a major public health and service issue [10].

The deeper concern is systemic: when a high-income, publicly financed system repeatedly intervenes only once disease becomes surgically ”efficient” to treat, care pathways may drift away from early prevention and toward acceptance of irreversible outcomes.

## 4. “Restoration Doesn’t Change the Fate”: Evidence, Misinterpretation, and the Service Context

UK practice-based research and subsequent trial programmes have repeatedly raised an uncomfortable question: does conventional restorative care, as commonly delivered in general practice settings under real-world constraints, reliably prevent pain, sepsis, and extraction?

The NIHR FiCTION programme and related publications explicitly emerged from uncertainty in primary care caries management. In the FiCTION three-arm RCT report, dental pain and/or dental sepsis occurred across all strategies, and modelling indicated no statistically significant differences between trial arms for the primary outcome when comparing strategies over follow-up [11]. This finding does not mean “restoration is pointless.” It means that within the system that delivered these strategies—with its access limits, follow-up variability, and behavioural constraints—no strategy can be treated as a magical substitute for early prevention plus reliable continuity of care.

A harmful misreading would be to treat these findings as justification for fatalism or for extraction-first policy (for example, implying that “primary teeth will be lost anyway”). The responsible interpretation is the opposite: when outcomes converge across strategies in real-world settings, it highlights the overriding influence of delivery conditions—access, continuity, incentives, and behavioural constraints. Improving these conditions is therefore a prerequisite for any clinical modality to realise its intended benefit.

## 5. The Hall Technique: From Pragmatic Innovation to System-Compatible Doctrine

### 5.1. What the Hall Technique Is (And Why It Spread)

Innes and colleagues described the Hall Technique as a simplified method using preformed metal crowns, cemented without local anaesthesia, with no caries removal and no tooth preparation [12]. Its behavioural and time advantages are obvious in pressured primary care.

Longer-term follow-up work reported that sealing caries using the Hall Technique outperformed “standard restorations” in that study context, with markedly lower failure rates [13].

### 5.2. The Uncomfortable Systems Question

The critique is not that the Hall Technique “does not work.” The critique is that systems under strain preferentially adopt interventions that minimise chair time and complexity, thereby risking elevating those interventions into default philosophies. When access is poor and follow-up is uncertain, “seal and survive” can become a defensible clinical stance. But when a wealthy system converts a scarcity-adapted technique into its baseline offer—without simultaneously restoring access and prevention capacity—it quietly redefines “evidence-based” as “system-compatible.”

In implementation terms, this is the central danger: efficacy can be reinterpreted into ideology when the service context is allowed to collapse.

It is important to acknowledge that Hall crowns and SDF have ethically sound “bridge-to-definitive-care” roles even in well-functioning services—for example, in very young children, in those with acute anxiety or neurodiversity-related tolerance limits, or where safeguarding and family instability make multi-visit operative care temporarily unrealistic. The systems critique here is not their use but the absence of protected recall, escalation, and definitive-care pathways that prevent temporisation from becoming the endpoint.

## 6. Silver Diamine Fluoride: A Valuable Tool That Can Become an Endpoint in a Broken Pathway

Silver diamine fluoride (SDF) is an important advance, particularly for disease control in very young children or those unable to tolerate conventional operative care. An umbrella review (Seifo et al.) reported that systematic reviews consistently supported SDF’s effectiveness in arresting caries (with black staining commonly reported as an adverse effect), while also noting limitations in the evidence for some prevention indications in children [14,15].

The system’s critique is again about use:

- In a functioning pathway, SDF should often be a **bridge** (pain prevention, risk reduction, stabilisation) while definitive care and sustained prevention are organised.
- In a constrained pathway, SDF risks being operationalised as **the last offer**, especially for disadvantaged children—turning an “advance” into a mechanism that absorbs pressure without fixing causes.

A wealthy system should not rely on pharmacologic arrest to compensate for the absence of access. It should use an arrest to *buy time* while making access real.

## 7. Water Fluoridation: Population Benefit, Evidence Nuance, and the Risk of Policy Overreach

### 7.1. Coverage Remains Limited

A Parliamentary POST brief (2024 update) states that fluoride is added via water fluoridation schemes to approximately 10% of the population in England [8].

### 7.2. Evidence Evolution: From York Caution to Contemporary Summaries

The University of York systematic review (McDonagh et al., 2000) found no randomised controlled trials of water fluoridation and highlighted methodological challenges and risks of confounding in observational comparisons [16].

More recently, an updated Cochrane review synthesising contemporary (post-1975) controlled observational evidence concluded that initiation of community water fluoridation may lead to a small reduction in dental caries in children, with effect estimates that include the possibility of little or no benefit, and reported insufficient evidence to determine impacts on socioeconomic inequalities [17]. This helps explain why policy summaries often endorse fluoridation as broadly effective while noting that modern effect sizes may be smaller and equity impacts uncertain [10].

### 7.3. The Key Implementation Point

Fluoridation is a risk modifier, not a substitute for care. No fluoridation scheme prevents the consequences of a child who cannot access a dentist until pain forces hospital referral. The policy error is treating population prevention as a substitute for service delivery reform. The correct stance is “both/and”: scale prevention and restore access.

## 8. MIH and Compromised First Permanent Molars: Guidance, Reality, and the Danger of Normalised Permanent Tooth Loss

The Royal College of Surgeons of England guideline on extraction of first permanent molars in children outlines MIH, the challenges of post-eruptive enamel breakdown and hypersensitivity, and the need for carefully timed, orthodontically informed case selection when prognosis is poor [18]. Importantly, the guideline explicitly acknowledges contextual factors such as service availability and the child’s capacity to receive complex care [14]. The guideline does not recommend indiscriminate extraction; the risk is how selective guidance is operationalised when capacity is constrained.

That contextual caveat is critical. Contemporary MIH management can be resource-intensive: repeated desensitisation and pain control, adhesive restorations with moisture control, stainless steel crowns for first permanent molars, and—where needed—sedation support and specialist paediatric/restorative input. When these capabilities and recall capacity are scarce, the treatment threshold shifts: the practical question becomes not “could this molar be saved?” but “can salvage be delivered and maintained within available pathways?”.

- In that environment, guidance intended for selective, prognosis-driven extraction can drift into an operational default because extraction is a single, schedulable endpoint whereas restoration requires multiple supported contacts. The consequence is that avoidable permanent tooth loss risks being normalised as an efficiency response to capacity constraints, with downstream occlusal and orthodontic consequences that are then managed rather than prevented.

An “advances” agenda must therefore pair MIH guidance with explicit capacity guarantees: timely access to clinicians experienced in MIH restorative care, defined referral and escalation pathways, and orthodontic assessment within the window required for favourable extraction planning. Otherwise, “planned loss” becomes a service workaround rather than a patient-centred choice.

## 9. What “Advance” Should Mean in NHS Paediatric Dentistry: Practical, System-Level Proposals

A credible advances framework for children’s dentistry in England should explicitly couple clinical tools with delivery guarantees. The following proposals add operational anchors, so recommendations are implementable rather than rhetorical:

1. Define unacceptable endpoints and publicly track them. Treat caries-related hospital tooth extraction episodes as preventable harms and review them as “sentinel events” rather than routine throughput. Practical trigger examples include the following: (a) multi-tooth caries-related hospital extraction in a young child; (b) repeat caries-related hospital extraction episodes within 24 months; and (c) caries-related hospital extraction following no documented preventive dental contact within the previous 12 months. OHID indicators can then be linked to prevention and access performance [7].
2. Rebuild the front door: access as a child health requirement. When survey data indicate that 96.9% of people without a dentist who attempted to access NHS dental care reported being unsuccessful [3,4], prevention guidance becomes aspirational for households. Commissioning should therefore specify and publicly monitor a child’s access standard (time-to-first appointment for new child patients) and protect urgent capacity so pain and infection do not become the default gateway.
3. Contract reform with prevention and complexity protected. NAO reporting underscores that recovery efforts sit on unresolved structural issues in how NHS dentistry is commissioned and delivered [6]. Any advances agenda that avoids contract and workforce reform—especially for time-intensive paediatric care, behaviour support, and safeguarding contexts—is incomplete.
4. Use biological approaches (Hall/SDF) with explicit “bridge rules”. Commission Hall crowns and SDF as stabilisation steps with (i) risk-based recall expectations (for high-risk children, typically 3–6 months), (ii) explicit escalation triggers (pain episodes, soft-tissue pathology, failed seal/crown, repeated SDF application without disease control), and (iii) a defined pathway to definitive care or specialist referral—so temporisation cannot become the endpoint [12,13,14].
5. Prevention at scale: fluoridation expansion plus targeted child programmes. Fluoridation currently reaches only a minority of England [10], and contemporary evidence suggests modest caries reductions with uncertainty around inequality impacts [17]. Combine fluoridation with supervised brushing, fluoride varnish programmes, and early-years integration, while avoiding framing population prevention as a substitute for clinical access and continuity [9].
6. MIH pathways must not become a euphemism for capacity limits. Define “multidisciplinary input” in practice (GDP plus paediatric/restorative assessment and timely orthodontic input) and ensure assessment occurs within the window needed for orthodontically favourable extraction planning. This helps keep elective extraction selective and prognosis-driven, rather than a default substitute for unavailable restorative capacity [18]. Table 1 contrasts the intended, pathway-based use of common paediatric dental interventions with their deployment under conditions of system constraint. It demonstrates how interventions designed as stabilising or complementary components of care may instead become default endpoints when access, workforce capacity, or service integration are limited, with implications for care quality and equity.

## 10. Conclusions

Paediatric dentistry in England does not lack clinical innovations. It lacks the consistent delivery conditions that allow innovations to function as intended. In a strained NHS dental system, “advances” can be repurposed into pressure valves: Hall crowns that become doctrine, SDF that becomes the last offer, fluoridation that becomes a political substitute for access reform, and MIH guidance that risks being operationalised as routine permanent tooth loss.

MDPI’s Collection “Advance in Pediatric Dentistry” is to be more than a catalogue of techniques; it must include a systems claim: If paediatric dental ”advances” are to fulfil their promise, they must be evaluated not only by clinical efficacy but by the delivery systems in which they operate. In a wealthy, publicly financed health system, sustained access, prevention at scale, and continuity of care are prerequisites for innovation to translate into improved outcomes. Without these conditions, even well-established advances risk being absorbed into pathways that prioritise manageability over long-term oral health.

## Figures and Tables

**Table 1:** Appropriate clinical use versus system-driven risk across common paediatric dental interventions.

Intervention	Appropriate Use in a Functioning Care Pathway	Risk When System Constraints Dominate
**Hall Technique (preformed metal crowns)**	Used as a pragmatic, evidence-based option to manage caries in primary molars where cooperation, tolerance, or behaviour limits conventional restorative care; embedded within a pathway that includes recall, prevention, and escalation where needed.	Becomes a default doctrine (“seal and survive”) selected primarily for speed and deliverability, with limited recall or progression to definitive care—functioning as an endpoint rather than a stabilising step.
**Silver diamine fluoride (SDF)**	Applied to arrest caries and reduce pain risk in young children or those unable to tolerate operative care, while access to definitive treatment and sustained prevention is actively organised.	Operationalised as the last offer in constrained pathways, particularly for disadvantaged children, absorbing system pressure without resolving underlying access or prevention failures.
**Extraction under general anaesthesia**	Reserved for cases where disease severity, age, behavioural capacity, safeguarding concerns, or acute infection make conservative care unsafe or unrealistic, and where earlier preventive and restorative opportunities have been pursued where possible.	Normalised as a routine endpoint for preventable disease due to delayed access and crisis-led presentation, rather than as a last-resort intervention within a continuum of care.
**Water fluoridation**	Implemented as a population-level risk modifier that complements individual preventive care and timely clinical access.	Framed as a substitute for service delivery reform, with population prevention expected to compensate for the limited reachability of routine dental care.
**MIH-related first permanent molar extraction**	Undertaken selectively, based on prognosis, orthodontic timing, and multidisciplinary assessment, where restorative salvage has been genuinely explored and deemed unsuitable.	Becomes an efficiency pathway when restorative capacity, recall infrastructure, or specialist access is constrained, narrowing clinical options and normalising avoidable permanent tooth loss.

## Data Availability

No new data were created or analysed in this study. All cited data are available in the referenced public sources.
